# Subject-specific measures of Achilles tendon moment arm using ultrasound and video-based motion capture

**DOI:** 10.1002/phy2.139

**Published:** 2013-11-07

**Authors:** Kurt Manal, Justin D Cowder, Thomas S Buchanan

**Affiliations:** 1 Department of Mechanical Engineering, Delaware Rehabilitation InstituteNewark, Delaware, 19716; 2 Department of Mechanical Engineering, University of DelawareNewark, Delaware, 19716

**Keywords:** Ankle joint, center of rotation, displacement, lever arm, tendon excursion

## Abstract

The Achilles tendon (AT) moment arm is an important biomechanical parameter most commonly estimated using one of two methods: (A) center of rotation and (B) tendon excursion. Conflicting findings regarding magnitude and whether it changes with contraction intensity have been reported when using these methods. In this study, we present an alternate method of measuring the AT moment arm by combining ultrasound and video-based motion capture. Moment arms for 10 healthy male subjects were measured at five different joint angles in 10° increments ranging from 20° of dorsiflexion (DF) to 20° of plantar flexion (PF). Moment arms were measured at rest and also during maximum voluntary contraction (MVC). For both conditions, the AT moment arm increased in magnitude as the ankle moved from DF to PF. In 20° of DF, the moment arm at rest averaged 34.6 ± 1.8 mm and increased to a maximum value of 36.9 ± 1.9 mm when plantar flexed to 10°. Moment arms during MVC ranged from 35.7 ± 1.8 mm to 38.1 ± 2.6 mm. The moment arms we obtained were much more consistent with literature values derived using ultrasound and tendon excursion compared to center of rotation or in vitro methods. This is noteworthy as the hybrid method is easy to implement and as it is less costly and timing consuming than other methods, including tendon excursion, it is well suited for large-scale studies involving many subjects.

## Introduction

Developing accurate estimates of musculotendon parameters is important for use in biomechanical studies and musculoskeletal models. The muscle moment arm is one such parameter; transforming the force developed by a muscle into a moment of force about a joint. The ankle plantar flexion (PF) moment is dominated by the powerful gastroc–soleus muscle complex with force development and timing integral to normal posture and ambulatory function. Although the uni-articular soleus and bi-articular gastrocnemii are functionally different, they both act to plantar flex the ankle via a common tendon (i.e., Achilles tendon). Accurate estimates of Achilles tendon (AT) moment arms are therefore important when investigating the force generating potential of the gastroc–soleus muscle complex and for studying function and energetics during normal and pathological gait.

The Achilles is the largest tendon in the human body and acts primarily in the sagittal plane causing the ankle to plantar flex about the talocrural joint. Several approaches have been used to estimate AT moment arm in vivo. These methods can be classified into two general categories: (A) center of rotation and (B) tendon excursion. The center of rotation method as applied to the AT is based on the method of Reuleaux (1963). The Reuleaux method uses X-ray or other imaging modalities such as magnetic resonance imaging (MRI) of the foot and tibia to indirectly approximate the ankle joint center, and from the image one can estimate the AT force vector as the midline of the tendon (Rugg et al. 1990; Maganaris et al. 1998, 2000; Maganaris 2004). The tendon excursion method derives the moment arm as the ratio of change in muscle-tendon length and joint angle (Grieve et al. 1978). The tendon excursion method for moment arm estimation can also be explained by the principle of virtual work (An et al. 1984).

Differing methodologies and sensitivities to measurement error raises the possibility that moment arms estimated using center of rotation and tendon excursion may yield different values even when applied to the same person. This was recently shown by Fath and colleagues who reported large differences (∼25%) between AT moment arms derived from tendon excursion compared to center of rotation (Fath et al. 2010). Moment arms for the MRI-based center of rotation were significantly larger than those obtained using ultrasound and tendon excursion. The authors did not opine about which method resulted in more anatomically plausible values; however, they did state that choice of method can have significant implications for musculoskeletal models. Maganaris et al. (1998, 2000) also reported differences in moment arm depending on which method was used, and to complicate matters, moment arms estimated using center of rotation changed in magnitude with level of contraction, while no changes were noted for tendon excursion.

We have developed a hybrid method of computing AT moment arm that leverages the strengths of ultrasound imaging and video-based motion capture to accurately resolve the moment arm directly at the joint angle of interest (Manal et al. 2010). The hybrid method has been shown to be accurate (3.3% error) based on testing using an animal surrogate of the AT tendon; the method, however, has not been tested with human subjects. The primary purpose of this study was to measure subject-specific AT moment arms in vivo using the hybrid method, and to evaluate if there is a change in magnitude between rest and maximum voluntary contraction (MVC). AT moment arms reported in the literature vary markedly and for this reason we sought to determine where along the continuum of values our data lie, and to explore if there is a trend in moment arm magnitude dependent on the particular methodology used.

## Material and Methods

**Figure 1:**
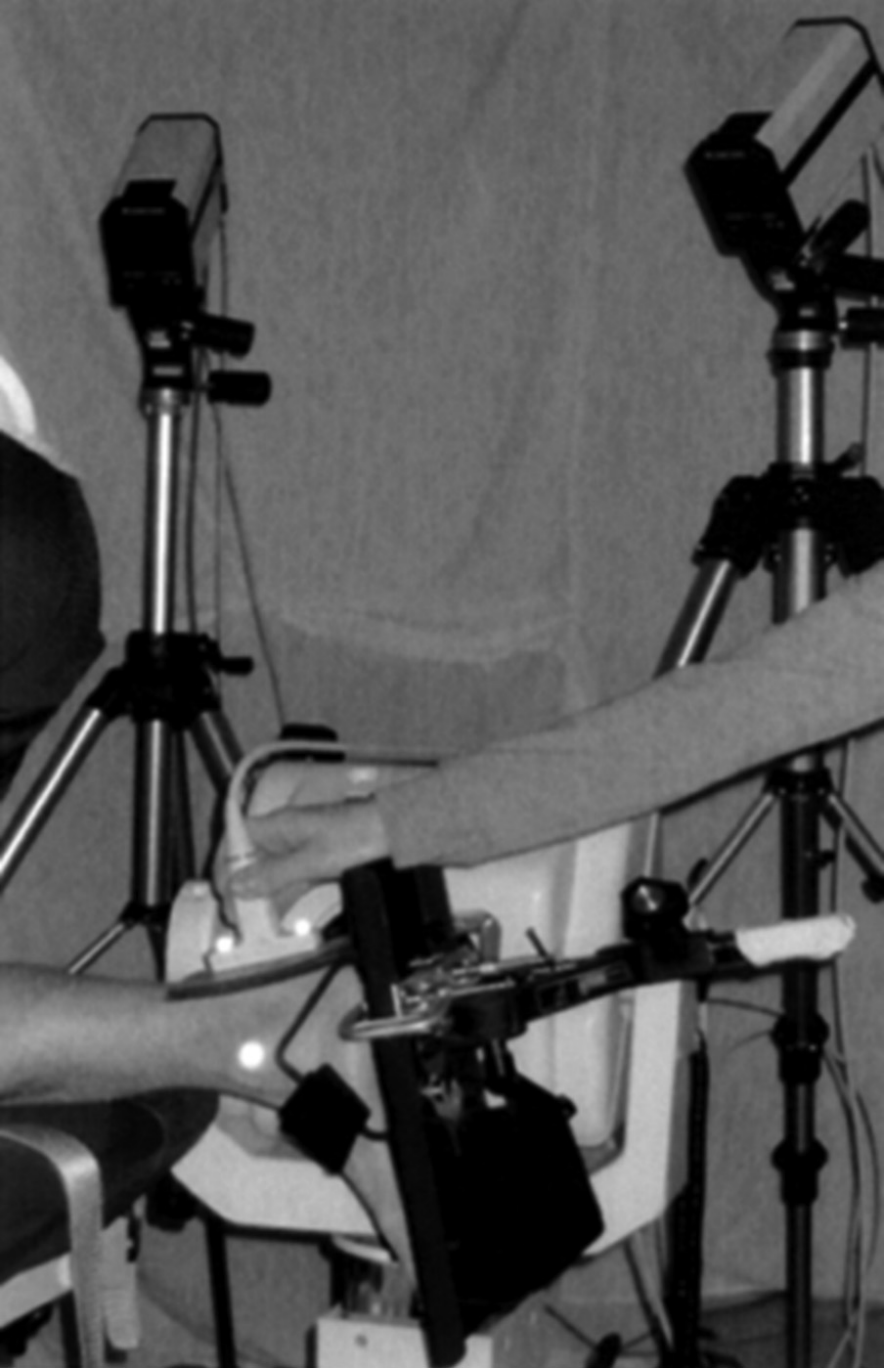
Subjects faced the back of the Biodex chair with the foot securely fastened to the foot plate. The ultrasound probe was positioned directly over the Achilles tendon and between the markers over the malleoli. Note the retroreflective markers on the casing of the ultrasound probe and use of gel pad during imaging.

Ten healthy adult male subjects participated in this study (average age: 24.1 ± 2.3 years, height: 1.77 ± 0.05 m and mass: 76.1 ± 9.1 kg). All subjects submitted written informed consent prior to testing and the testing protocol was approved by the Human Subjects Review Board at the University of Delaware. Subjects were placed in a Biodex System 3 isokinetic dynamometer (Biodex, Shirley, NY) in a reverse seated position with the torso resting against the back of the chair (Fig. 1). The subject's left foot was secured to the foot plate attachment of the dynamometer using Velcro straps. A rigid foam restraint was placed between the subject's knee and chair back to minimize heel liftoff during MVC. The foam block also helped maintain the knee in approximately 90° of flexion throughout testing. The dynamometer was used to determine ankle joint range of motion, monitor joint angle during testing, and to provide resistance during isometric PF MVC. A gel standoff pad was placed over the posterior aspect of the distal shank and heel to maximize acoustic coupling during imaging of the AT (Fig. 1). Only the left leg was tested.

**Figure 2:**
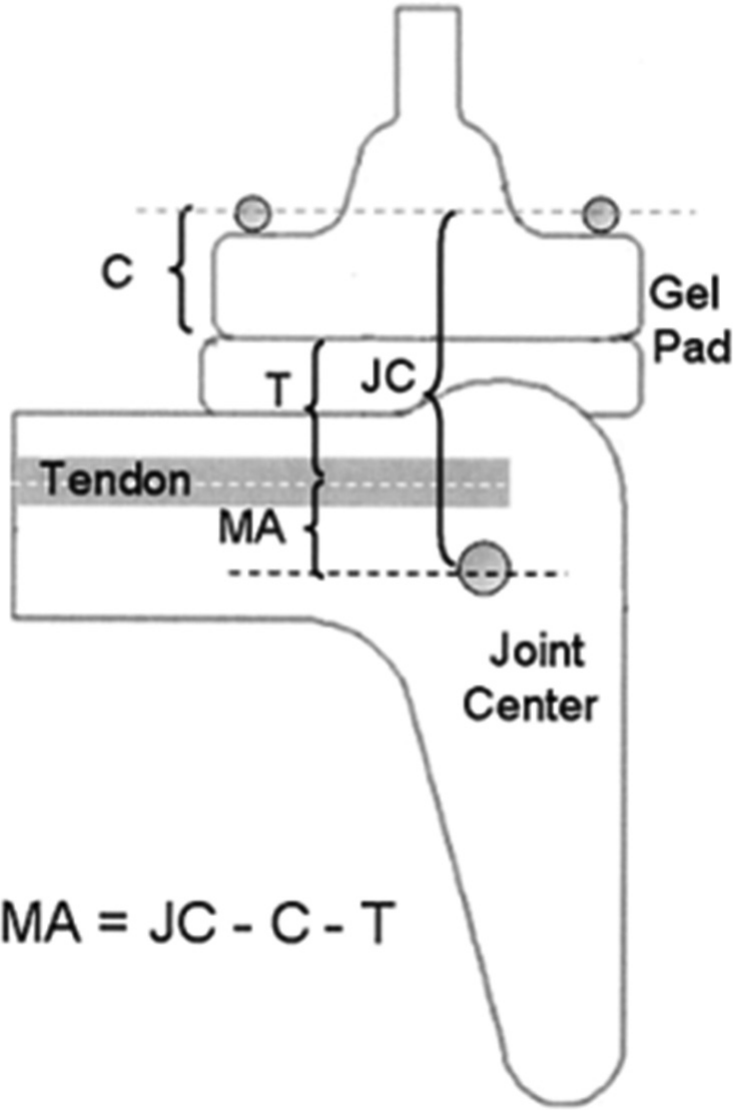
A typical sonogram of the Achilles tendon. The distance or depth from the top of the image to the midline of the tendon (black-dashed line) was measured using the ultrasound console software. In this example, the distance was 12.9 mm. Where along the top of the sonogram to measure the depth is determined in a separate process using markers on the ultrasound probe and over the malleoli. For illustrative purposes the location is indicated by the white circle with black dot, while in reality the distance from the right edge of the sonogram is measured with the ultrasound system software. Refer to Manal et al. (2010), for details.

Achilles tendon moment arms were computed using a previously described hybrid method (Manal et al. 2010) and therefore the approach will only be described briefly. A seven camera motion capture system (Qualisys ProReflex, Gothenburg, Sweden) was used to track retroreflective markers positioned over both malleoli at a sampling rate of 30 Hz. The ankle joint center was assumed to lie at the midpoint between the malleoli as commonly done in gait studies (Kadaba et al. 1990; Davis et al. 1991). In addition, two retroreflective markers were placed on the back of the US probe directly over the limits of the field of view of the transducer (see Fig. 2). The markers on the probe provided a spatial correspondence between the three-dimensional position of the ankle joint center in a fixed laboratory reference and the location of the joint center in the sonogram. Sagittal plane B-mode imaging of the AT was acquired using a 60-mm linear probe with an ultrasonic frequency of 10 MHz (Aloka SSD-5000, Tokyo, Japan). Motion capture data and US images were synchronized using a pressure-sensitive contact switch taped directly over the acquire image button on the US console. When the acquire image button was depressed there was a corresponding time-synchronized 5 V square wave generated by the contact switch. The square wave and motion capture data were both sampled at 30 Hz allowing us to determine exactly what marker data to associate with the US image. Ultrasound images were saved in DICOM format and processed offline. A schematic of the moment arm calculation is illustrated in Figure 2.

Measurements were recorded in 10-degree increments ranging from 20° of dorsiflexion (DF) to 20° of plantar flexion. Four trials were completed at each joint angle tested: two trials at rest and two at MVC. For all trials, the US probe was aligned along the direction of the AT and centered between the markers over the malleoli. Data were collected for 7 sec for all trials (rest and MVC). During MVC trials subjects were instructed to develop and hold an isometric PF effort by pushing as hard as possible against the footplate of the dynamometer. The contraction was held for up to 5 sec to ensure an optimal US image. The MVC and resting trials were collected in an alternating manner providing subjects with approximately 2 min of rest between MVC exertions. Two-way analysis of variance (ANOVA) with repeated measures was used to compare MAs at five different joint angles (−20, −10, 0, 10, and 20) and for two levels of contraction (rest and MVC). An alpha level of 0.05 was used to evaluate statistical significance, and dependent *t*-tests with Bonferroni correction were used for post hoc testing when differences were detected.

## Results

**Figure 3:**
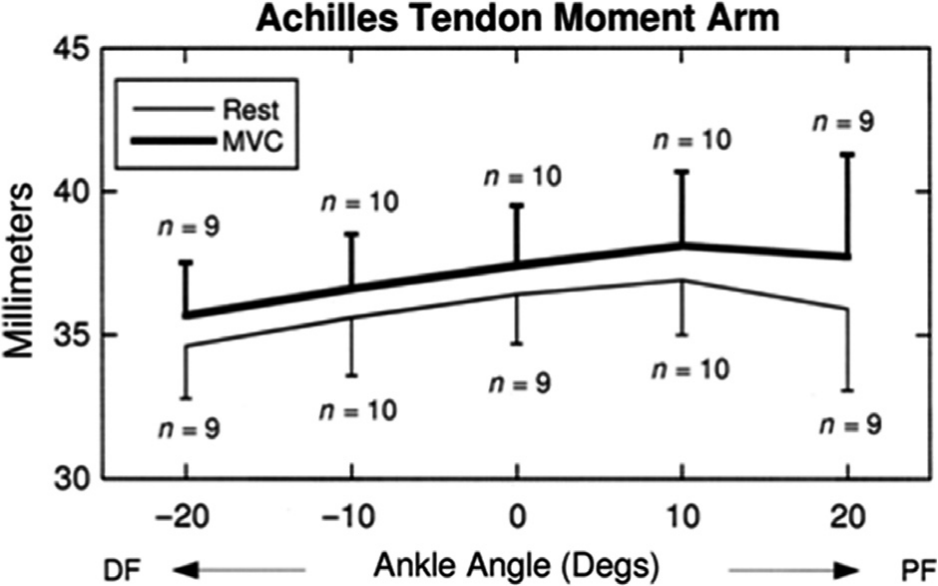
Achilles tendon MAs for all subjects with average values reported in millimeters and standard deviation bars. Note the trend of increasing moment arm with PF and that only a small increase in magnitude was observed between rest and MVC. Also included in the figure is the number of subjects for whom complete data were available at each joint angle.

Moment arms at rest ranged from 34.6 ± 1.8 mm when dorsiflexed 20° to a peak value of 36.9 ± 1.9 mm in 10° of PF (Table 1). Moment arms increased slightly during MVC ranging from 35.7 ± 1.8 mm to 38.1 ± 2.6 mm over the same ankle joint range of motion. The smallest moment arm recorded was 30.9 mm during rest with the ankle in 20° of DF and the largest value was 43.1 mm in 20° of PF during MVC (N. B., these were not for the same subject). The average increase from rest to MVC over the range of angles tested was only 3.4 ± 0.9% (Fig. 3). Included in Figure 3 is the number of subjects for each of the joint angles tested. Data for one subject was missing because he could not reach 20° of DF (peak DF was 14°), whereas data for two others could not be used because a marker over the malleoli was obscured during data acquisition (Table 1).

**Table 1:** Achilles tendon moment arms at rest and during maximum voluntary contraction

	Achilles tendon moment arm				
	
	20° DF	10° DF	Neutral	10° DF	20° DF
Rest	34.6 (1.8)	35.6 (2.0)	36.4 (1.7)	36.9 (1.9)	35.9 (2.8)
MVC	35.7 (1.8)	35.6 (1.9)	37.4 (2.1)	38.1 (2.6)	37.7 (3.6)

Average values and (standard deviations) are reported in millimeters.

There were no statistical differences in moment arm magnitude between rest and MVC nor was the interaction of angle and contraction intensity significant. There was a main effect of angle after applying the Greenhouse-Geisser correction *F*(1.12, 5.91) = 6.60, *P* < 0.05. Post hoc testing revealed the moment arm at 20° DF was significantly smaller compared to moment arms at 10°DF, neutral (i.e., 0°) and 10° PF. The Bonferroni corrected level of significance for these comparisons was *P* < 0.005.

## Discussion

The primary purpose of this study was to apply the hybrid method of measuring the AT moment arm in vivo and to evaluate if moment arm magnitude changed with level of contraction. Moment arms were measured over a functional range of motion (20° DF to 20° plantar flexion) as it encompasses normal ankle motion during walking (Perry 1992).

A very consistent trend of increasing moment arm with plantarflexion was observed for all subjects at rest and also during MVC. This finding is consistent with several other studies (Rugg et al. 1990; Hintermann et al. 1994; Maganaris et al. 1998, 2000; Maganaris 2004). We also noted that there was little change in magnitude between rest and MVC across all ankle angles tested. The difference was small with an approximate 3% increase during MVC when averaged across subjects over the range of angles tested. This finding is similar to observations by Maganaris et al. (1998, 2000) who also reported no change in magnitude between rest and MVC when the moment arm was estimated using tendon excursion. The implication is that if the magnitude does not change with contraction intensity then this would greatly simplify modeling efforts as a single value for the moment arm could be used at a given joint angle for contractions ranging from 0% to 100% MVC.

The AT moment arm is an important biomechanical parameter and one that varies markedly between studies. Literature values across the joint angles we tested span a range from approximately 30 mm in DF (Hintermann et al. 1994) to as much as 70 mm in PF (Maganaris 2004). Subject stature is a confounding factor when comparing between studies and for this reason we recruited our subjects by sex and stature to facilitate comparisons with the widely cited data of Maganaris and colleagues. Our 10 male subjects were 24.1 ± 2.3 years, 1.77 ± 0.05 m tall, and weighed 76.07 ± 9.07 kg. This agreed closely with the demographics of their six male subjects (28 ± 4 years, height 1.75 ± 0.08 m, and mass: 75 ± 7 kg). The average peak moment arm we measured was 38.1 mm during MVC with the ankle in 10° of PF. In contrast, Maganaris et al. (2000)reported a value of approximately 52 mm at the same joint angle when using tendon excursion. Because the subjects were matched by stature, differences between our values and theirs were most likely related to methodological differences rather than subject variability. To further illustrate this point consider that when center of rotation was applied to exactly the same six subjects the moment arm increased from 52 mm to 64 mm Maganaris et al. 2000 These results highlight the sensitivity of the AT moment arm to the particular methodology used. Inman commented on using the Reuleaux method to locate the ankle joint center. In his words: “The accuracy of the results using the method of instantaneous centers of rotation depends on the number and closeness of the points of least motion” (Inman 1976). Maganaris used a rather large angular increment of 15° which may explain, in part, why moment arms for exactly the same group of subjects were strikingly different when estimated using tendon excursion compared to center of rotation methods (Maganaris et al. 1998, 2000).

Two recent studies highlight the influence that different methods can have when estimating the AT moment arm. For example, using tendon excursion Fath et al. (2010), reported a value of approximately 36 mm with the ankle in 15° of PF, whereas the moment arm for the same subjects was 55 mm with center of rotation. Hashizume et al. (2012) reported relatively large differences in moment arm when comparing 2D versus 3D approaches. Moment arms for the 2D center of rotation were significantly larger than 3D estimates based on a finite helical angle approach (see Table 2). These studies in addition to work by Maganaris and colleagues clearly show that different moment arms are obtained depending on the particular methodology used. This is an important point when comparing results and interpreting findings between studies in which different methods were implemented.

**Table 2:** Survey of AT moment arms reported in the literature

Achilles tendon moment arms						

Study	Modality	Method	Subjects	Contraction	Angle (PF)	MA (mm)
Fath et al. (2010)	MRI	COR	7 M, 2 F	Rest	15	55.4
Fath et al. (2010)	US	TE	7 M, 2 F	Rest	15	36.2
Hashizume et al. (2012)	MRI	COR	15 M	Rest	10	53
Hashizume et al. (2012)	MRI	3D FHA	15 M	Rest	10	41.4
Hintermann et al. (1994)	In vitro	TE	8 M, 7 F	N/A	10	∼ 52
Kawakami et al. (2001)	US	TE	6 M	Submax	10	∼ 40
Lee and Piazza (2009)*	US	TE	24 (M + F)	Rest	10 DF – 20 PF	∼ 36
Maganaris et al. (2000)	MRI	COR	6 M	Rest	10	∼ 51
Maganaris et al. (2000)	MRI	COR	6 M	MVC	10	∼ 64
Maganaris et al. (2000)	US	TE	6 M	Rest	10	∼ 53
Maganaris et al. (2000)	US	TE	6 M	MVC	10	∼ 53
Manal (this study)	US	Hybrid	10 M	Rest	10	36.9
Manal (this study)	US	Hybrid	10 M	MVC	10	38.1
Rosager (2002)	US	TE	10 M	Submax	Neutral	∼ 56
Rugg et al. (1990)	MRI	COR	10 M	Submax	10	∼ 57
Sheehan (2012)**	MRI	IHA	19 (M + F)	Submax	10	∼ 53
Spoor et al. (1990)	In vitro	TE	2 M	N/A	10	∼ 51

A reference angle of 10° PF was used whenever possible. The “∼” indicates the moment arm was approximated from a figure in the relevant publication. COR, center of rotation; TE, tendon excursion; FHA, finite helical angle; IHA, instantaneous helical angle.

* Lee and Piazza reported an average moment arm over the range reported in the table.

** Unscaled values reported by Sheehan.

We noted an interesting and noteworthy trend when comparing AT moment arms derived from in vitro experiments and those based on MR images compared to moment arms determined from ultrasound. Using 10° of PF as reference angle, ultrasound-based estimates, including our values at rest and MVC, averaged approximately 36 mm, (Kawakami et al. 2001; Lee and Piazza 2009; Fath et al. 2010), whereas MRI and in vitro estimates averaged more than 52 mm (Rugg et al. 1990; Spoor et al. 1990; Hintermann et al. 1994; Klein et al. 1996; Maganaris et al. 1998, 2000; Maganaris 2004; Fath et al. 2010; Sheehan 2012). All of the latter studies reported moment arms greater than 50 mm while none of the ultrasound-based measures were greater than 40 mm (Table 2). Moment arms were clearly and consistently clustered in magnitude depending on methodology. This dependency is important to recognize and clinically relevant. For example, inaccurate estimates of moment arm could impact surgical planning and procedures for patients with musculoskeletal pathology (Arnold et al. 2000). Furthermore, a muscle with a long moment arm will undergo a greater amount of shortening and it will shorten at a greater velocity for a given joint rotation relative to a muscle with a shorter moment arm (Nagano and Komura 2003; Lee and Piazza 2009). It follows from the force–velocity relationship that the muscle will develop less force when contracting concentrically compared to a muscle with the shorter moment arm. The ankle plantar flexors function concentrically during late stance when the PF moment is largest. Inaccurate estimates of AT moment arm not only affect muscle force estimates but also energetic measures such a joint work and power, integral to the study of normal and pathological gait mechanics.

As with all new methodologies it is important to evaluate the reliability of the technique. Using a very similar approach combining US and marker data to quantify AT length, Silbernagel et al. (2012) found that test–retest reliability was excellent (ICC = 0.97, 95% confidence interval = 0.86–0.99) and there were no significant differences (*P* = 0.889) between the two test occasions. Although we have not conducted a rigorous reliability evaluation of the hybrid method, we have compared test–retest values for three subjects over separate days and found an average difference of 4.3% at rest and 5.6% during MVC. Taken together, these data suggest that the hybrid method may be a reliable technique and an appropriate method when subjects are tested on multiple occasions.

Subject positioning is an important consideration when using dynamometry and great effort was taken to ensure proper alignment between the trans-malleolar axis and the axis of rotation of the dynamometer. The axes were considered aligned when markers over the malleoli moved minimally relative to the head of the dynamometer as the ankle was moved through its range of motion during subject positioning. Once aligned, we assumed that the ankle joint center remained relatively fixed during testing as visually the malleolar markers did not appear to move appreciably. Although this assumption was not required of our method, it implies that a change in moment arm was associated with a tendon shift rather than a change in the location of the ankle joint center. It was evident from the ultrasound images that the tendon moved upward toward the top of the sonogram (i.e., a posterior shift relative to the joint center) during the MVC trials compared to the midline of the tendon at rest. Overall, the posterior shit was quite small, on the order of several millimeters, which was approximately the difference in moment arm between rest and MVC.

**Figure 4:**
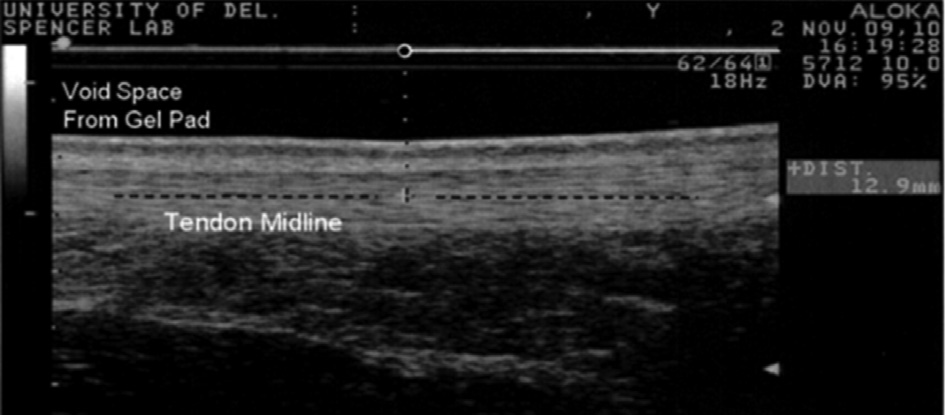
Sagittal plane schematic of the method for computing Achilles tendon moment arm. MA, moment arm; C, constant distance from line between markers on probe to recording surface of the ultrasound transducer; JC, perpendicular distance from line between markers on probe and the ankle joint center approximated from markers over the malleoli; T, distance from bottom of transducer to tendon midline. A gel pad was used to improve acoustic coupling. Note: the method was depicted in 2D for illustrative purposes; the resulting moment arm is the 3D distance from the line of action of the Achilles tendon to the 3D location of the ankle joint center.

There are several limitations of our method that should be considered. First, defining the ankle joint axis as we did requires placement of reflective markers over the malleoli. Misplacing the markers will introduce errors in the location of the joint center and subsequently the moment arm. Misplacing the markers too far forward over the malleoli will result in a moment arm that is too large, and vice versa. We attempted to minimize such errors by positioning the markers over the most prominent portion of each malleoli and thereby standardizing placement. Another potential source of error when using our method is the subjective nature of identifying the midline of the tendon on the sonogram. We believe this represents only a small source of error; however, since even in the worst case scenario it is difficult to misidentify the midline of the tendon by more than a millimeter assuming a good quality ultrasound image. From Figure 4 one can see that misidentifying the midline of the tendon by 1 mm will result in a 1 mm error in the value of the moment arm. Transducer placement and applied pressure are also potential sources of errors. Misaligning the ultrasound probe sufficiently to cause a projection error with significant effect on the moment arm is difficult as the line of action of the AT is easily visualized facilitating correct probe alignment. It is possible that the tendon was restricted from shifting posterior relative to the ankle joint center due to pressure applied by the experimenter when positioning the US probe. The experimenter did his best (same experimenter for all subjects) to apply minimal and consistent pressure during testing. Some pressure, however, was required to ensure good contact for quality of the US image. The actual force applied was not quantified and we assumed that it was similar within and between subjects. A possible effect of this applied force would be a reduction in moment arm, and thus the values we obtained may represent a lower limit for individuals of similar sex and stature as the subjects we tested. The center of rotation and tendon excursion methods are not subject to this limitation as there is no direct application of pressure to the back of the lower leg during testing. Consequently, the AT can shift posterior relative to the joint center while contracting and there will be a positive linear increase in the moment arm when estimated using center of rotation. In contrast, moment arms derived from tendon excursion are generally based on displacement of the myotendinous junction which acts primarily along the long axis of the musculotendinous unit. Thus, if the AT did shift posterior relative to the joint center it would only have a minimal affect on the longitudinal displacement of the myotendinous junction and consequently only a small influence on the moment arm.

The hybrid approach outlined in this study is easy to implement, combining two reliable and valid measurement instruments: video-based motion capture and ultrasound imaging. The method has been tested using an animal surrogate and shown to have good accuracy (3.3% error) and indirect evidence suggests that the method is reliable. The moment arms we obtained were similar in magnitude to values reported by others using tendon excursion derived from US. This is noteworthy as the hybrid method is easier to implement and less time consuming and thus it can be applied to many subjects facilitating the development of subject-specific models on a large-scale basis. A unique aspect of the technique is that it measures the moment arm directly at the joint angle of interest. This is relevant as moment arms derived using tendon excursion and ultrasound have been shown to exhibit a rotational dependency. That is, moment arms calculated as the ankle moved from DF to PF were different from values obtained when the ankle was moved from PF to DF (Fath et al. 2010). The method we developed is not subject to this rotational dependency as the moment arm is measured directly at the angle interest. Tendon compliance is known to change with age (Stenroth et al. 2012), and to our knowledge there have been no studies evaluating the effect of tendon compliance on moment arms computed using tendon excursion. Older tendon is more compliant than young and therefore it is possible that the myotendinous junction of an older subject will displace a greater amount and consequently the moment arm will be larger compared to a younger subject of identical stature. The hybrid measurement is not influenced by tendon compliance and therefore it may be an ideal approach when comparing moment arms for young and older subjects.

There is a growing interest in developing subject-specific models for use with biomechanical studies. The ability to measure the AT moment arm in a timely and cost-efficient manner is a precursor to achieving this goal. The hybrid method is one such approach. The moment arms we obtained were similar in magnitude to values derived from tendon excursion and ultrasound. This is noteworthy as the hybrid method is easier to implement and thus may be well suited for large-scale studies. Finally, moment arms reported in the literature appear to be clustered in magnitude depending on the specific methodology. The hybrid method and studies using tendon excursion and US tend to yield smaller values than in vitro studies and those using center of rotation. This point is important to consider when comparing moment arms between studies and interpreting findings in cases where different methodologies have been used to determine the moment arm.
